# Detection of antibodies against flavivirus over time in wild non-human primates from the lowlands of Costa Rica

**DOI:** 10.1371/journal.pone.0219271

**Published:** 2019-07-05

**Authors:** Gaby Dolz, Andrea Chaves, Gustavo A. Gutiérrez-Espeleta, Edgar Ortiz-Malavasi, Sofía Bernal-Valle, Marco Vinicio Herrero

**Affiliations:** 1 Laboratorio de Entomología, Programa de Investigación en Medicina Poblacional, Escuela de Medicina Veterinaria, Universidad Nacional, Heredia, Costa Rica; 2 Escuela de Biología, Universidad de Costa Rica, San José, Costa Rica; 3 Escuela de Forestales, Instituto Tecnológico de Costa Rica, Cartago, Costa Rica; Institut Pasteur of Shanghai Chinese Academy of Sciences, CHINA

## Abstract

Two-hundred-nine free ranging non-human primates from 31 locations throughout Costa Rica were captured and released between 1993 and 2012, and blood samples, sera or plasma were collected, to detect antigens and antibodies, and so assess the distribution of active and passive flavivirus infections over time. A competitive enzyme-linked immunoassay for the detection of antibodies was used to determine the distribution of past flavivirus infections over time, while Reverse Transcriptase Polymerase Chain Reaction (RT-PCR) was used to detect active West Nile Virus (WNV) and Dengue virus (DENV) infections. The first serological evidence of flavivirus in these animals was determined in 1993, at the same time when DENV re-emerged in humans from Costa Rica. An increase in the number of seropositive wild monkeys to flavivirus was determined over time in the country (11.3% seropositivity in 1993–1996, 20.7% in 2001–2008, and finally 52.9% in 2010–2012). Furthermore, the presence of DENV2 was detected in samples from four howler monkeys collected in 2001–2002, whereas DENV2, DENV3, and DENV4 were found in samples from four white-faced monkeys, and WNV in three howler monkeys living in the Pacific coast of Costa Rica during 2010–2012. The habitat where the positive PCR individuals lived were characterized as fragmented forests, having temperatures ranging from 26°C to 28°C, altitudes below 250 meters above sea level, high precipitation during 7 to 9 months (1500–4000 mm), and a marked dry season of 3 to 5 months. All these animals were living near mangroves; however, they did not show clinical signs of illness at the time of sampling. Results obtained show that the number of seropositive wild non-human primates to flavivirus were increasing during time in the country, longitudinal studies are needed to investigate their role as sentinels of these viruses and to determine if flavivirus infections can affect these species.

## Introduction

West Nile virus (WNV) and Dengue virus (DENV) have been classified within the genus Flavivirus, family Flaviviridae, and are part of the medically important Japanese encephalitis virus (JEV) serocomplex [1]. Each of these viruses causes similar disease syndromes in humans, manifesting as an asymptomatic or mild flu like illness to clinical encephalitis [2]. Eradication of the vector *Aedes aegypti* was certified in 1955 in Costa Rica, and after decades of absence, DENV was reintroduced in 1993, the year in which the first endogenous transmissions were reported in the country [3]. Since then, the four serotypes (DENV1 to DENV4) are co-circulating, showing epidemic peaks every three years with low mortality (0.3 per 100000 habitants), as compared worldwide. The cases were diagnosed initially on the Pacific coast and extended progressively to the rest of the country [4]. West Nile Virus was introduced in the Northeastern United States in 1999 and gradually spread across the continent. Countries of Latin America with reported activity for West Nile virus between 2001 and 2004, included Mexico, Belize, Guatemala, El Salvador, Colombia, Venezuela, and Argentina [5, 6, 7, 8]. The first case of WNV in Costa Rica was reported in 2009 in a horse from the Guanacaste region with encephalitis [9]. Although WNV was reported to cause mortality among equines and certain domestic and wild birds [10], human infections in endemic areas are usually mild or subclinical, severe disease is commonly associated with the elderly [11].

Flavivirus infection can be detected within 4 to 7 days after initial exposure by IgM capture enzyme-linked immunosorbent assay (ELISA), or by indirect IgG-ELISA within 8 days after the onset of symptoms. The development of WNV competitive ELISA had the advantage, in that it measures the ability of antibodies present in sera to block the binding of a monoclonal antibody to protein E, beyond that, it is species independent, and detects flavivirus antibodies in several species of domestic mammals, that may persist for more than one year. Since flavivirus-infected sera shows cross-reactions with heterologous flavivirus infections such as WNV, DENV, JEV, and other members of this serocomplex [2, 12], the plaque reduction neutralization test (PRNT) is still used as the reference assay for specific diagnosis of flavivirus infection [13]. However, PRNT is a laborious test and uses live virus. Although virus isolation in cell culture is the current method of choice for the detection of WNV in vertebrate tissues, reverse transcriptase polymerase chain reaction (RT-PCR) has been used to develop highly sensitive and specific assays for the identification of WNV [14].

To date, most of the studies of flavivirus have focused on humans, arthropods and domestic and laboratory animals. However, DENV and WNV have an extremely wide host range among vertebrates [15, 16]. The role of some of these other species in the maintenance and amplification of DENV and WNV remains to be determined. Non-human primates can sustain viral replication of DENV in relevant cell types and develop a robust immune response, but they do not develop overt disease [17]. With respect to WNV, activity was demonstrated in a non-human primate breeding colony and in the neighboring community during the same period [18]. The high infection rate in this captive non-human primate population showed that transmission can occur silently in nature among susceptible vertebrates during epidemic periods [18]. For this reason, they can be used as sentinels for the presence of those viruses. Brazil is the only neotropical country reporting antibodies against flavivirus in two species of non-human primates tested [19]. Costa Rica has four non-human primate species distributed in different regions of the country and all have declined sharply in both the original distribution as well as abundance. Currently, the spider monkey (*Ateles geoffroyi*) is classified as endangered; the squirrel monkey (*Saimiri oerstedii*) is classified as a vulnerable species, while the white-faced monkey (*Cebus imitator*) and howler monkey (*Alouatta palliata*) are classified as least concern [20].

The objective of the present study was to determine antibodies or RNA in plasma and blood samples from wild non-human primates collected between 1993 and 2012 in the lowlands of Costa Rica, in order to assess the distribution and seropositivity of flavivirus infections over time.

## Materials and methods

### Study area, sampling size and sampling

The present study was conducted with protocols approved by the Comisión de Bienestar Animal, Universidad Nacional, Heredia, and adhered to the legal requirements of Costa Rica.

Thirty-one locations throughout the country were chosen opportunistically, based on the sighting of monkey troops, regardless of the species present, and in locations representative of habitats of these animals. Three locations were visited twice during the investigation. The geographic coordinates of each location were obtained using a GPS unit (Table 1). The land relief and the main climatic characteristics (elevation, temperature, precipitation, number of rainy days and dry months) were determined with data obtained from the Costa Rican Atlas 2014 (Centro Científico Tropical 2013) (Table 1).

**Table 1:** Attributes of climatic zones of the study areas of non-human primates, Costa Rica.

Site	Location	Latitude	Longitude	Elevation (MASL)	Mean Temperature (°C)	Precipitation (mm)	# rainy days	#dry months
1	La Pacífica, Cañas	10°27'3.71"N	85° 7'37.52"O	100	26–28	< 1500	150	6
2	Río Jesús, San Ramón	10° 1'43.83"N	84°31'5.99"O	400	22–24	4000–5000	190	5
3	Finca Jiménez Núñez, San Ramón	10° 5'28.88"N	84°28'59.63"O	1230	22–24	2000–3000	190	5
4	Earth, Guácimo	10°10'42.91"N	83°36'53.48"O	32	22–24	3000–4000	365	1
5	Limón, Limón	9°59'21.05"N	83° 1'59.43"O	27	26–28	3000–4000	330	1
6	Chomes, Puntarenas	10° 2'37.87"N	84°54'29.67"O	22	> 28	1500–2000	150	5
7	Bebedero, Cañas	10°22'7.62"N	85°11'45.17"O	39	26–28	1500–2000	190	5
8	Palo Verde, Bagaces	10°23'0.29"N	85°20'3.47"O	127	> 28	1500–2000	150	5
9	Cahuita, Talamanca	9°44'5.23"N	82°50'42.77"O	0	26–28	3000–4000	270	1
10	Copal, Nicoya	10°10'8.89"N	85°15'49.82"O	70	> 28	1500–2000	170	5
11	Puerto Jesús, Nicoya	10° 6'40.59"N	85°16'10.78"O	20	> 28	1500–2000	190	5
12	El Palmar, Puntarenas	10° 1'36.70"N	84°46'23.17"O	9	26–28	1500–2000	170	5
13	Manuel Antonio, Quepos	9°24'37.76"N	84° 9'9.34"O	0	26–28	3000–4000	250	3
14	Nogal, Sarapiquí	10°27'41.85"N	83°58'0.14"O	38	24–26	3000–4000	330	1
15	Pacuare, Turrialba	9°54'17.76"N	83°39'55.05"O	1600	22–24	2000–3000	250	2
16	La Catalina, Siquirres	10° 9'0.19"N	83°31'8.02"O	10	24–26	3000–4000	350	1
17	Carara, Garabito	9°42'6.73"N	84°32'37.05"O	100	26–28	1500–2000	210	4
18	Samay, Pococí	10°43'28.90"N	83°34'41.86"O	0	26–28	5000–6000	350	1
19	Conchal, Santa Cruz	10°23'57.47"N	85°48'15.73"O	0	26–28	4000–5000	250	3
20	Playa Naranjo, Liberia	10°46'30.77"N	85°39'46.85"O	0	26–28	< 1500	70	6
21	Finca Barú, Quepos	9°27'37.11"N	84° 7'48.31"O	100	26–28	3000–4000	250	3
22	Sierpe, Osa	8°50'52.15"N	83°30'0.18"O	0	26–28	4000–5000	250	3
23	San Joaquín, Puntarenas	10° 0'15.84"N	84°43'20.93"O	100	26–28	1500–2000	190	5
24	Caño Negro, Los Chiles	10°53'32.68"N	84°47'44.41"O	100	26–28	4000–5000	230	3
25	Puerto Vargas, Talamanca	9°43'42.18"N	82°49'5.51"O	0	26–28	3000–4000	270	1
26	Linda Vista, Pococí	10°25'29.57"N	83°43'28.39"O	100	26–28	4000–5000	365	1
27	Pueblo Nuevo, Pococí	10°19'54.72"N	83°35'48.56"O	100	24–26	3000–4000	365	1
28	Naranjito, Quepos	9°28'14.25"N	84° 6'14.13"O	100	26–28	4000–5000	250	3
29	Ostional, Santa Cruz	10° 1'15.09"N	85°43'15.97"O	0	26–28	2000–3000	210	4
30	Nosara, Nicoya	9°58'46.00"N	85°38'55.55"O	0	26–28	2000–3000	210	4
31	La Virgen, Sarapiquí	10°24'5.15"N	84° 8'3.41"O	200	24–28	4000–5000	290	1

MASL: Meters above the sea level

The National System of Conservation Areas (SINAC) from the Ministry of Environment and Energy (MINAE) issued the permit for the field work in the seven national parks (Palo Verde, Cahuita, Manuel Antonio, Carara, Playa Naranjo, Caño Negro, Puerto Vargas). The remaining locations were private land, where owners gave permission to carry out field studies. A total of 71 plasma samples from howler monkey were collected during the period between 1993 to 1996 from three locations, and were subjected only to serological analysis, whereas 87 plasma samples (82 howler monkeys, 2 squirrel monkeys, and 3 white-faced monkeys) were collected between 2001 and 2008 from 14 locations and were analyzed by serological (87) and molecular (84) techniques. Finally, 71 whole blood and serum samples (30 howler monkeys, 31 white-faced monkeys, 8 squirrel monkeys, and 2 spider monkeys) were collected between 2010 and 2012 from 17 different locations, and were analyzed by serological (51), and molecular (71) techniques, respectively (Table 2).

**Table 2:** Results of wild non-human primates analyzed for antigens and antibodies against flavivirus and antigens at various locations of Costa Rica during the period between 1993 and 2012.

Period	Site	PCR+/Total	Seropositives/Total	Species	Gender	Age
					Female (F), Male (M)	Adult (A), Juvenile (J), Infant (I)
**1993–1996**	1	ND	3/52	Howler monkey	ND	ND
	2	ND	1/7	Howler monkey	ND	ND
	3	ND	4/12	Howler monkey	ND	ND
**Total**		** N/D**	**8/71 **			
**2001–2008**	4	0/3	2/3	Howler monkey	ND	ND
	3	0/3	1/3	White-faced monkey	1 M, 2 ND	1 A, 2 ND
		0/4	0/4	Howler monkey	1 F, 3 M	1 A, 3 ND
	5	0/2	1/3	Spider monkey	3 F	3 A
	6	0/9	3/9	Howler monkey	5 F, 4 M	3 A, 6 ND
	7	0/2	0/2	Howler monkey	1 F, 1 M	ND
	8	4/16^1^	2/16	Howler monkey	6 F, 10 M	2 A, 14 ND
	9	0/29	5/29	Howler monkey	18 F, 10 M, 1 ND	4 A, 25 ND
	10	0/3	1/3	Howler monkey	2 F, 1 M	ND
	11	0/2	1/2	Howler monkey	1 F, 1 M	ND
	12	0/4	0/4	Howler monkey	3 F, 1 M	ND
	13	ND	1/2	Squirrel monkey	ND	ND
	14	0/1	0/1	Howler monkey	F	ND
	15	0/1	0/1	Howler monkey	M	J
	16	0/5	1/5	Howler monkey	4 F, 1 M	1 A, 1 J, 3 ND
**Total**	** **	**4/84**	**18/87**		** **	** **
**2010–2012**	17	0/9	7/9	White-faced monkey	4 F, 5 M	7 A, 2 J
		0/2	1/2	Howler monkey	1 F, 1 M	1 A, 1 J
	13	0/2	0/2	White-faced monkey	1 F, 1 M	1 A, 1 J
		0/4	1/4	Howler monkey	3 F, 1 M	4 A
		0/4	0/1	Squirrel monkey	2 F, 2 M	4 A
	18	0/3	1/3	Howler monkey	1 F, 2 M	3 A
	19	0/6	3/6	Howler monkey	4 F, 2 M	5A, 1 J
	20	0/2	2/2	White-faced monkey	1 F, 1 M	2 A
	21	2/6^2^	5/6	White-faced monkey	3 F, 3 M	6 A
	22	2/2^3^	1/2	White-faced monkey	2 M	1 J, 1 A
	23	0/7	5/7	White-faced monkey	1 F, 5 M, 1 ND	5 A, 2 I
	24	0/1	1/1	White-faced monkey	M	A
		0/1	0/1	Howler monkey	ND	ND
	1	0/2	0/2	Howler monkey	2 F	1 A, 1 I
	25	0/2	0/2	White-faced monkey	2 M	2 A
	26	0/1	0/1	Spider monkey	M	A
	27	0/3	ND	Howler monkey	1 F, 2 M	2 A, 1 J
	28	0/4	ND	Squirrel monkey	3 F, 1 M	2 A, 2 J
	29	1/3^4^	ND	Howler monkey	3 F	3 A
	30	2/4^4^	ND	Howler monkey	3 F, 1 M	4 A
	31	0/2	ND	Howler monkey	1 F, 1 M	2 A
		0/1	ND	Spider monkey	1 F	1 A
**Total**	** **	**7/71**	**27/51**		** **	** **

ND: Not determined

^1^PCR positives to DENV2

^2^PCR positives to DENV1, DENV3, DENV4

^3^PCR positives to DENV2, DENV4

^4^PCR positives to WNV

The animals were captured *in situ* by chemical immobilization with darts (Type P, 1ml, Pneu Dart Inc., Williamsport, PA, USA), and compressed gas rifle (X-Caliber Gauged CO2, Pneu Dart Inc.) for individuals over long distances, or blowgun for individuals in close proximity. Anesthetics used were Zoletil 50 (3.3-11mg/kg) or ketamine (5-20mg/kg), in combination with xylazine (0.5-2mg/kg) [21, 22]. As soon as the animal was anesthetized, it was safely caught in a nylon net, and assessed by a veterinarian that performed a physical exam, took the blood sample and accompanied the monkey until it was fully recovered from anesthesia, and then it was returned to its natural habitat, at the same location of capture. In some cases, information about gender and age (adult or juvenile) was recorded. The blood sample (2-4ml) was taken from the femoral, saphene or cephalic vein, and collected in tubes with and without EDTA, and maintained at 4°C until arrival to the laboratory, where samples were separated, and whole blood, plasma and sera were kept at -20°C until analysis.

### Serological and molecular surveys

A total of 209 samples were tested with the IDScreen West Nile Competition Multi-species (ID-Vet Innovative Diagnostics, Montpellier, France), a competitive ELISA for the detection of antibodies directed against protein E of WNV, as well as other viruses of the JEV serocomplex. The kit was used according to the manufacturer´s instructions. Briefly, 50μL of samples and control sera were mixed with an equal volume of dilution buffer and added to the wells, that were pre-coated with recombinant protein E of WNV, during 90 min at room temperature (RT). After washing the plates 3 times, 100μL of a monoclonal antibody (anti-protein E of WNV) conjugated to horseradish peroxidase was added to the wells during 30 minutes at RT, and plates were washed again three times. After addition of 100μL of substrate solution during 15 minutes at RT, reaction was stopped with 100μL of stop solution and the plates read with a spectrophotometer at 450 nm. Results were validated to determine if the mean of the optical density (OD) of the negative control was >0.7, and the mean value of the positive control OD was <30% of the negative control OD. Inhibition percentages (I%) were calculated using the optical density of tested serum samples and the average of the optical density of the negative control sera, using the formula: I% = (OD of sample × 100): (average OD of negative control). As recommended by the manufacturer, serum samples with I% ≤40% were considered as positive; samples with I% between 40 and 50% were scored as inconclusive (negative in the present study), and sera with values >50% were determined to be negative.

All 84 plasma and 71 blood samples were extracted using QIAamp Viral RNA Mini Kit (Qiagen, Hilden) according to the manufacturer’s instructions. The RT-PCR was carried out for DENV (DENV1 to DENV4) and WNV as described previously using highly specific primers and sensitive conditions [23, 24]. Briefly, the conventional semi-nested protocol for serotyping DENV as described by Lanciotti et al. [23], that amplifies a segment of *C-prM* region was used. For detection of WNV the RNA was subjected to a RT-PCR as described by Lanciotti et al. [24] that amplifies a region of the WNV envelope gene. The DENV-positive controls used in the RT-PCR were DENV 1 to 4, kindly donated by the Dengue Reference Center, Instituto Costarricense de Investigación y Enseñanza en Nutrición y Salud (INCIENSA), Cartago, Costa Rica. The WNV-positive control was RNA purchased at National Veterinary Services Laboratories, United States Department of Agriculture (NVSL-USDA), Ames, Iowa, USA. Free nuclease water was used as negative control. Reverse transcriptase PCR products were visualized using a 2% agarose gel electrophoresis stained with Gel Red. Samples that showed bands with the expected size (DENV1: 482bp, DENV2: 119bp, DENV3: 290bp, DENV4: 392bp, WNV: 400bp) were considered positive.

## Results

A total of 53 (25.4%) out of 209 individuals yielded positive results in ELISA, while only two animals (1.0%) showed inconclusive reactions. Most of the positive (94.7%), and negative (98.8%) reacting sera and plasma of non-human primates showed inhibition percentages far away from the cut off values of the competitive ELISA. As shown in Table 3, sera determined as positive in the competitive ELISA in the three sampling periods showed mean inhibition percentages between 8.5%-18.4%, with maximum values between 19.4 and 38.2 and P75 (that is, the I% of 75% of the sera tested) in ranges 10.3%-29.2% (Table 3).

**Table 3:** Serological statistical values obtained with the West Nile Competition Multi-species ELISA testing wild non-human primates (NHP) of Costa Rica between 1993 and 2012.

	NHP sera collected between 1993–1996	NHP sera collected between 2001–2008	NHP sera collected between 2010–2012
Inhibition values	Positive reacting sera	Negative reacting sera	Positive reacting sera	Negative reacting sera	Positive reacting sera	Negative reacting sera
Mean I% (%)	10.8	82.0	18.4	94.3	8.5	89.5
Min.	5.0	42.0	4.6	76.5	2.5	76.0
Max.	25.0	106.0	38.2	119.6	19.4	100.1
P 25 (%)	7.8	78.5	7.6	90.2	4.9	84.8
P 75 (%)	12.0	85.0	29.2	97.6	10.3	95.2

**Mean I%: Average inhibition percentage; Min: Minimum inhibition value; Max: maximum inhibition value; P25: 25**^**th**^ **percentile of inhibition values; P75: 75**^**th**^ **percentile of inhibition values Mean I%:** mean inhibition percentage determined for positive and negative sera, **Min.**: Minimum Inhibition value determined for positive and negative sera, **Max**.: Maximum Inhibition value determined for positive and negative sera, P25: 25^th^ percentile, that is, the Inhibition percentage of 25% of the sera tested, P75: 75^th^ percentile, that is, the Inhibition percentage of 75% of the sera tested

**Fig 1:**
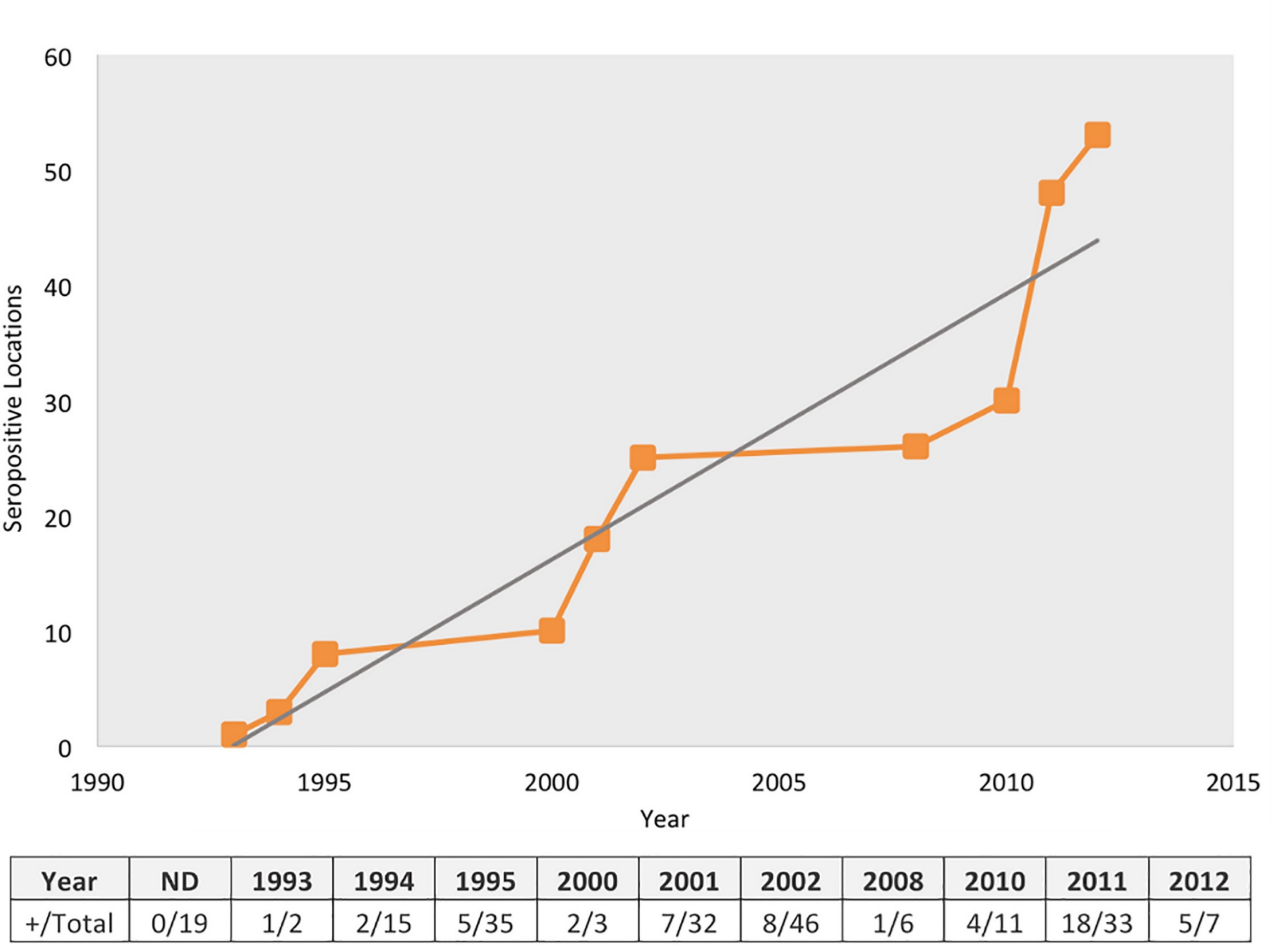
Cumulative curve of locations with seropositive wild non-human primates to flavivirus over time. ND: year not determined.

An increase in the number of seropositive wild monkeys to flavivirus was determined over time at sites with wild NHP (Fig 1).

**Fig 2:**
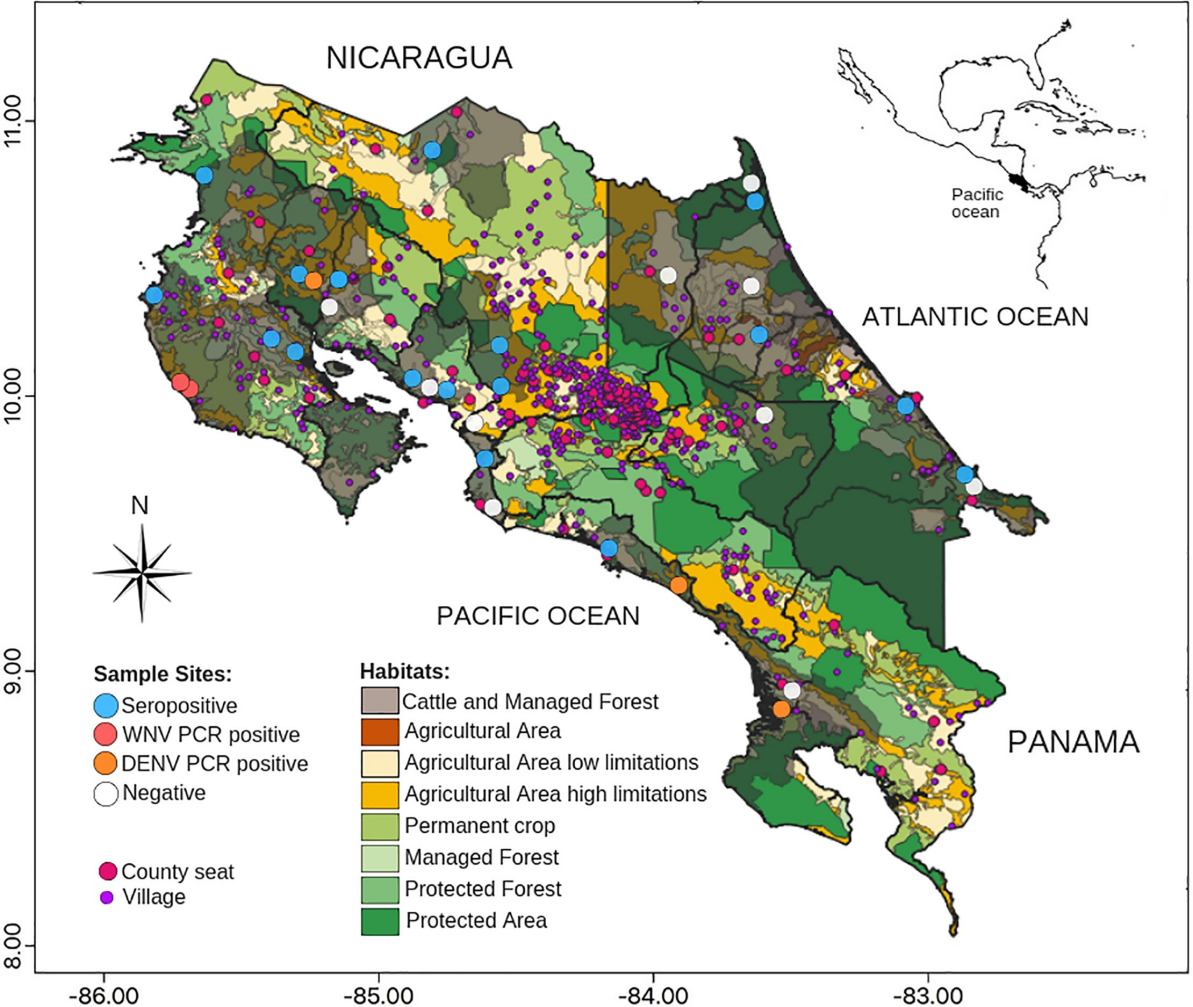
Sites with wild non-human primates seropositive to flavivirus, and PCR positives to DENV and WNV, 1993–2012 in Costa Rica. Reprinted from Atlas de Costa Rica under a CC BY license, with permission from Edgar Ortiz Malavasi, original copyright 2014.

Eight (11.3%) out of 71 plasma samples from howler monkeys collected in the period between 1993 to 1996 showed antibodies against protein E, whereas two (2.8%) individuals showed inconclusive reactions. Seropositive reacting howler monkeys to flavivirus were detected in all three-survey sites between 1993 and 1996 (Table 2, Fig 2). Gender and age were not recorded from these seropositive animals.

Antibodies against flavivirus were detected in 18 of 87 (20.7%) samples from monkeys collected between 2001 and 2008. Seropositive individuals to flavivirus were detected in ten (71.4%) out of 14 sample points (Table 2, Fig 2). Seropositive individuals were howler monkeys, one white-faced monkey, one spider monkey and one squirrel monkey (Table 2), ten were females, four males, and in four animals’ gender was not recorded. A total of four samples (4.6%) of howler monkeys collected in this period time (2001–2002) in Palo Verde, in the Northern Pacific Coast (site 8), were positive to DENV2 in RT-PCR, only one of these animals, a female, also showed positive results in ELISA, whereas the other three male monkeys were seronegative.

From the 51 serum samples collected between 2010 and 2012 a total of 27 (52.9%) yielded positive results in ELISA. The positive individuals were identified in nine (75.0%) different locations out of 12 sites analyzed (Table 2, Fig 2), whereas in five sampling sites, sera were not collected, and presence of antibodies not determined (Table 2). The six seropositive howler monkeys were four males and two females, all adult animals, whereas the 21 seropositive white-faced monkeys were represented by 13 males and eight females, most were adults (18) and three juvenile animals. A total of four (5.6%) out of 71 blood samples yielded positive results in PCR to DENV2 (one white-faced monkey), DENV3 (one white-faced monkey) and DENV4 (two white-faced monkeys). Of these, two white-faced monkeys (one female and one male, both adults) from Finca Barú (site 21) were positive, one to DENV3, and the other to DENV4, respectively, whereas two white-faced male adult monkeys from Sierpe (site 22, further South from site 21) were positive, one to DENV2 and the other to DENV4. Animals PCR positive to DENV4 were seronegative, whereas individual’s PCR positive to DENV2 and DENV3 showed antibodies against protein E in ELISA. Furthermore, three samples (4.2%) yielded positive results in RT-PCR to WNV, these animals were two howler monkeys (adult male and female) captured in Ostional (site 30) and one adult female in Nosara (site 29) in the Northern Pacific coast. Serum was not available from these animals.

## Discussion

These results represent the first report of serological detection of flavivirus in non-human primates from Costa Rica. The first serological evidence of flavivirus in these animals was determined in 1993, at the same time when DENV re-emerged in humans from Costa Rica [3]. Most probably the antibodies detected against protein E in animals during the first sampling period (1993 to 1996) were due to DENV infection, since WNV was introduced first in the American continent in 1999, and no other flavivirus was reported during these years in Costa Rica. The increase of seropositivity to flavivirus in wild monkeys during that time is in accordance with the spreading of DENV, and probably afterwards, with the introduction of WNV, throughout the country [3]. Although serological assays establish past infections [16], DENV2 active infections were determined with RT-PCR between 2001 and 2002 in four howler monkeys from Palo Verde, in the Northern Pacific coast, a non-fragmented forest, with temperatures > 28°C, located at an altitude 1500–2000 meters above sea level, having precipitations in ranges of 1500–2000 mm, and a marked dry season of five months. This National Park is classified as a wetland, which implies a high mosquito density, and might explain the active DENV infections detected at this site. Only one of these animals showed antibodies to flavivirus in ELISA. A possible explanation for this might be that this animal was presenting an acute infection or had previous contact with another DENV serotype or flavivirus that did produce antibodies; however, these antibodies did not prevent active infection with DENV2. Also, two troops of white-faced monkeys from the Southern Pacific coast from Costa Rica showed simultaneously presence of two serotypes of DENV in 2011, confirming the co-circulation of various serotypes of DENV and its wide distribution in the country [3]. In Sierpe, only two animals from the troop were sampled, one animal showing active infection with DENV4, and no antibodies against flavivirus protein E, whereas another individual showed an active infection with DENV2 and a positive serological reaction. The same was established in the white-faced monkey troop from Finca Barú. Five out of six animals showed positive reactions in ELISA; indicating, that these animals were exposed before to DENV or another flavivirus, the monkey with active infection of DENV4 had however no antibodies. These troops were captured on sites with secondary forest moderately fragmented, and close to water bodies, showing temperatures in ranges of 26–28°C, located at altitudes 0–100 meters above sea level, having precipitations in ranges of 2000–3000 mm, and a marked dry season of three months.

Active infections of WNV were detected in howler monkeys near Ostional and Nosara beaches, at the Northern Pacific coast in Guanacaste in 2010, and are in accordance with reports of equine WNV cases made by Jiménez et al. [9]. These sites with active cycles of WNV were characterized by low land forest close to mangrove and secondary forest, highly fragmented, having temperatures ranging from 26°C to 28°C, an altitude below 25 meters above sea level, precipitation for 210 days (2000–3000 mm), and a marked dry season of four months.

Although the serological assay used in the present assay had the disadvantage that it could not differentiate infections caused by heterologous flavivirus, it was established that most of the positive (94.7%) and negative (98.8%) reacting sera, and plasma of non-human primates showed competition values in this assay far away from the cut off value, so that it is unlikely that false positive or negative reactions occurred. So, this ELISA seems to be useful for monitoring flavivirus infections in populations where transmission can occur silently, for retrospective analysis, and for analyzing a large number of samples, since it is not as laborious and needs no viable virus [2, 12, 14, 18].

The present study showed that sites with seropositive wild non-human primates to flavivirus are increasing over time in our country. However, since sampling was not carried out longitudinally in the same places, it is also possible that places that were sampled later had higher prevalence’s. Animals diagnosed as PCR positive to DENV and WNV did not show clinical signs of illness. Further longitudinal studies are needed in order to confirm the increase of seropositivity to flavivirus in wild monkeys over time to investigate the consequences of these infections in NHP, and to establish their role as sentinels in DENV and WNV epidemiological cycles.

## Supporting information

S1 Table Complete database of sera and plasma of wild non-human primates (NHP) collected in Costa Rica between 1993 and 2012 and analyzed for flavivirus. (XLSX)
